# On the Accuracy and Scalability of Probabilistic Data Linkage Over the Brazilian 114 Million Cohort

**DOI:** 10.1109/JBHI.2018.2796941

**Published:** 2018-02-16

**Authors:** Robespierre Pita, Clĺcia Pinto, Samila Sena, Rosemeire Fiaccone, Leila Amorim, Sandra Reis, Mauricio L. Barreto, Spiros Denaxas, Marcos Ennes Barreto

**Affiliations:** 1 R. Pita and C. Pinto are with the Institute of Mathematics and Statistics, Computer Science Department, Federal University of Bahia, Salvador 40170-115, Brazil (e-mail: pierre.pita@ufba.br; cliciasp@ufba.br); 2 S. Sena, R. Fiaccone, and L. Amorim are with the Institute of Mathematics and Statistics, Department of Statistics, Federal University of Bahia, Salvador 40170-115, Brazil (e-mail: mylasenna@ufba.br; fiaccone@ufba.br; leiladen@ufba.br); 3 S. Reis and M. L. Barreto are with the Centre for Data and Knowledge Integration for Health (CIDACS), Oswaldo Cruz Foundation, Salvador 41940-220, Brazil (e-mail: ssreis@bahia.fiocruz.br; mauricio.barreto@bahia.fiocruz.br); 4 S. Denaxas is with the Institute of Health Informatics, University College London, London WC1E 6BT, U.K. (e-mail: s.denaxas@ucl.ac.uk); 5 M. E. Barreto is with the Institute of Mathematics and Statistics, Computer Science Department, Federal University of Bahia, Salvador 40170115, Brazil, and also with the Institute of Health Informatics, University College London, London WC1E 6BT, U.K. (e-mail: marcosb@ufba.br)

**Keywords:** Data linkage, accuracy assessment, cohort study

## Abstract

Data linkage refers to the process of identifying and linking records that refer to the same entity across multiple heterogeneous data sources. This method has been widely utilized across scientific domains, including public health where records from clinical, administrative, and other surveillance databases are aggregated and used for research, decision making, and assessment of public policies. When a common set of unique identifiers does not exist across sources, probabilistic linkage approaches are used to link records using a combination of attributes. These methods require a careful choice of comparison attributes as well as similarity metrics and cutoff values to decide if a given pair of records matches or not and for assessing the accuracy of the results. In large, complex datasets, linking and assessing accuracy can be challenging due to the volume and complexity of the data, the absence of a gold standard, and the challenges associated with manually reviewing a very large number of record matches. In this paper, we present AtyImo, a hybrid probabilistic linkage tool optimized for high accuracy and scalability in massive data sets. We describe the implementation details around anonymization, blocking, deterministic and probabilistic linkage, and accuracy assessment. We present results from linking a large population-based cohort of 114 million individuals in Brazil to public health and administrative databases for research. In controlled and real scenarios, we observed high accuracy of results: 93%-97% true matches. In terms of scalability, we present AtyImo’s ability to link the entire cohort in less than nine days using Spark and scaling up to 20 million records in less than 12s over heterogeneous (CPU+GPU) architectures.

## I Introduction

**D**ATA linkage is a widely adopted technique for combining data from disparate heterogeneous sources potentially belonging to the same entity [1], [2]. It has been applied in several domains to aggregate data to be used in decision-making processes, monitoring and surveillance tasks, assessment of public policies, and clinical research [3], [4].

In the context of public health, we linked data from a very large socioeconomic cohort consisting of 114 million individuals who have received payments from a conditional cash transfer program in Brazil between 2007 and 2015 to records from public health databases. We generated bespoke data sets for research studies aiming to quantify and evaluate the impact of such payments on several disease outcomes.

Besides data volume, the complexity of our scenario comes from the absence of common key attributes in all databases involved. This imposes the use of probabilistic approaches which, in turn, have a strong requirement on accuracy. Another challenging issue is the lack of gold standards to validate these linkages, as the amount of cohort participants appearing in any health database is unknown.

This is an extended version of our award-winning poster presented at IEEE Biomedical and Health Informatics 2017 [5]. In this paper, we present our data linkage tool (AtyImo) and its pipeline structure for anonymization, block construction, and pairwise comparison. AtyImo implements a mixture of deterministic and probabilistic routines for data linkage. We discuss and evaluate accuracy, scalability, and performance results achieved in experimental and real scenarios.

This paper is organized as follows: Section II presents some related work on data linkage tools and accuracy assessment issues. Section III presents the AtyImo tool and describes its functionalities. We provide a summary of our case study in Section IV. Different accuracy and scalability results are presented and discussed in Section V, and some conclusions and ideas for further research are presented in Section VI.

## II Related Work

Data linkage is implemented in vendor-specific databases and analytics platforms, statistical software, and research-centered solutions. In this section, we list some existing tools relying on some form of block construction and probabilistic technique to enable data linkage. Additionally, we discuss the accuracy assessment process and its associated challenges.

### A Data Linkage Tools

Reclink [6] provides different matching and block construction routines to support data linkage. Phonetic codes are applied over linkage attributes to generate candidate blocks for pairwise comparison. It was used in some ecological and small-size (nearly 5.700 individuals) cohort-based studies using Brazilian governmental data, such as [7] and [8].

Merge Toolbox [9] is offered by the German Record Linkage Center together with other tools for privacy-preserving matching (Safelink) and error imputation for accuracy validation (TD-Gen). FRIL [10] offers an interactive linkage process allowing users to select comparison attributes, a similarity function, and a decision model to accept or reject matched records. Febrl [11] is an open-source tool with a graphical interface that allows the combination of different encoding, indexing, comparison, and classification functions.

HARRA [12] and NC-Link [13] are proposals focused on machine learning techniques to perform record classification of large-scale data sets. Machine learning-driven approaches are also used in [14] to classify clusters of records generated by the MFIBlocks algorithm for uncertain multientity resolution, as well in [15] for classifying online customer profiling data. Different metablocking algorithms to entity resolution are discussed in [16], with emphasis on load balancing, graph (block) construction, and entity comparison.

A data mining platform targeting health care data is presented in [17]. It employs Apache Drill to support schemaless access to diverse data sources. The authors claim that this platform shortens the time needed to make data available for analysis when compared to other existing tools, presenting runtime performance results for *join* and *distinct* queries.

In [18], parallel data linkage algorithms and performance results obtained with data sets scaled up to 6 million records are discussed. Further, in [19], a Web-based version of these algorithms is compared against Febrl and FRIL. Privacy-preserving linkage methods implemented in OpenCL are discussed in [20], with emphasis on block construction and similarity calculation. Different blocking and clustering techniques to scale record linkage methods are discussed in [21]. Hybrid architectures were used in [22] to evaluate linkage performance over NVIDIA and OpenCL, resulting on a speedup of ten times for a 1.7 million data set from *freedb.org*.

### B Accuracy of Data Linkage

Common approaches for accuracy assessment comprise of the following:

1. the usage of “gold standards” (when true match status is known);
2. sensitivity analysis based on different linkage criteria;
3. comparison between linked and nonlinked records; and
4. statistical techniques dealing with uncertainty and bias measurement [23].

The entire scope of this topic also comprises proposals dealing with data quality and preparation, multiple imputation problems, bias and uncertainty quantification, as well scalability modeling [24].

When gold standards are absent, one must rely on controlled experiments with small size databases from which we can perform a manual review on linked records to quantify the accuracy and then scale to bigger databases. Accuracy can be measured through sensitivity, specificity, positive predictive value (PPV), and receiver operating characteristic (ROC) curves, as discussed in [25]–[27].

An alternative approach to assessing the accuracy is to utilize machine learning techniques for automating the process of tuning the linkage hyperparameters and reduce or eliminate the amount of human intervention. This approach is discussed in [28] and delivers highly accurate results from unsupervised methods as compared to existing gold standards. In [29], a discussion is provided on the manner that artificial neural networks and clustering algorithms can be used to deal with missing data and produce accurate results.

Our work contributes to the field by providing a scalable tool capable of linking very large databases with complex relationships and great variability in terms of data quality. Other contributions arise from the discussion on metrics for accuracy assessment, reference cutoff values and establishment of gold standards for probabilistic linkage.

## III AtyImo Data Linkage Tool

We initially developed AtyImo in 2013 to serve as a linkage tool supporting a joint Brazil–U.K. project aiming at building a large population-based cohort with data from more than 100 million participants and producing disease-specific data to facilitate diverse epidemiological research studies. The volume and heterogeneity of the databases involved, as well the absence of common key attributes among them and the expected cohort size (initially 80 million records) have posed strong requirements on scalability and accuracy. To address these challenges, we designed and implemented AtyImo as a modular pipeline, encapsulating components for data preprocessing, pairwise comparison, and matching decision.

Prior to linkage, all input data sets pass through a data quality analysis stage which performs data integrity and missingness checks, which quantify the percentage of missing data especially from linkage attributes. Any required procedures for data cleansing are also applied in this stage. The goals are to identify the suitability of linkage attributes (given missing data statistics) and recover records presenting some imputation errors that can be fixed through standardization procedures. The processes have been implemented using a variety of statistical analysis tools such as Stata and R.

### A Data Preprocessing

This stage is responsible for data harmonization, blocking, and anonymization. Common operations for data harmonization comprise date and string formatting, removal of special characters, and insertion of specific values for missing data.

Blocking [23] is a common approach used in data linkage that consists of grouping candidate pairs with similar characteristics to be subsequently compared. It reduces the execution time, as only similar blocks (and not all existing ones) are compared at a time. Blocking additionally helps to overcome space (memory) limitations when dealing with large databases.

Different techniques can be used for blocking: single key, predicates or machine learning-driven methods. Single key blocking is simpler, as only one attribute is used to group records, but errors in this key attribute can prevent a given record to be inserted into a block, thus resisting comparison with potential pairs. A refined approach consists of combining several key attributes into a disjunctive predicate used to correctly block records even if some of these attributes have errors. Finally, classification algorithms can use specific rules to learn how effectively and accurately construct blocks.

**Fig. 1:**
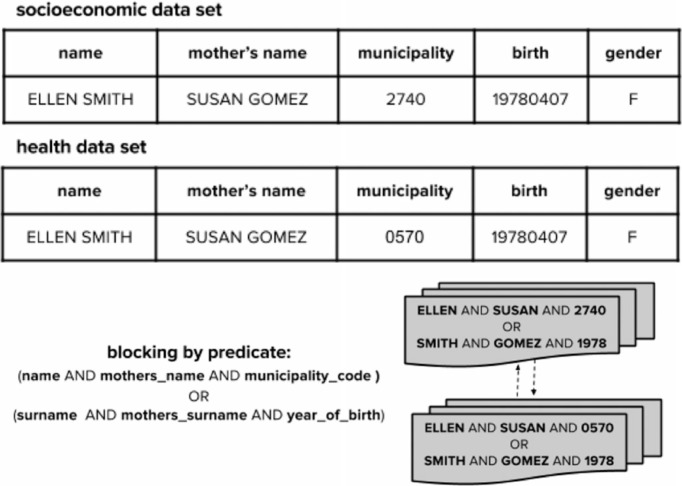
Blocking predicate implemented by AtyImo.

Fig. 1 shows the predicate-based blocking strategy used in AtyImo. After analyzing different predicates, we have chosen the following one: (*name AND mother_name AND mu-nicipality_code*) *OR* (*surname AND mother_surname AND year_of_birth*). It guarantees that errors in one clause do not prevent the record to be correctly grouped, resulting in a relatively small number of blocks of moderate sizes.

**Fig. 2:**
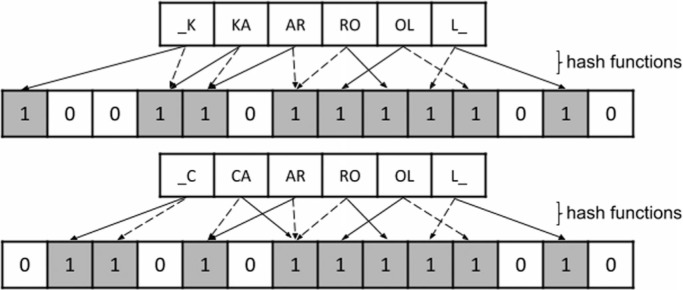
Example of a Bloom filter encoding hashed bigrams.

Anonymization is a critical issue for health data and different privacy-preserving techniques can be used to address this problem [30]–[32]. AtyImo uses Bloom filters [33], which are binary vectors of size *n* initialized with 0 (zero). Linkage attributes being anonymized are decomposed in “bigrams” (pairs of characters, including spaces) processed by hash functions to determine which positions in the filter must change to 1, as depicted in Fig. 2. The amount of positions depends on each attribute’s weight. Bloom filters are very reliable as two identical set of attributes will always generate the same vector [no false positives (FPs)]. After evaluating different configurations, we defined a 180-bit filter built from two hash functions and the following attributes (and weights): *name* and *mother_name* (50 bits each), *date_of_birth* (40 bits), *municipality_code,* and *gender* (20 bits each).

### B Pairwise Comparison Methods

**Fig. 3:**
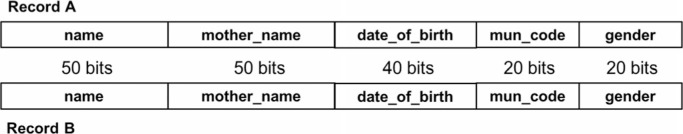
Full probabilistic linkage approach comparing Bloom filters directly.

AtyImo provides two approaches for pairwise comparison. The first one is *full probabilistic,* in which Bloom filters representing linkage attributes are entirely compared (see Fig. 3). A similarity value is calculated based on the Sørensen–Dice index [34], defined as Dice = (2 * *h*)/(*a* + 6), being *h* the total of 1’s at the same positions in both filters, and *a* and *b* the total of 1’s in the first and second filters, respectively. A Dice = 1 means filters are completely equal, decreasing to 0 (zero) depending on existing differences. Our implementation normalizes Dice indices between 0 and 10.000.

**Fig. 4:**
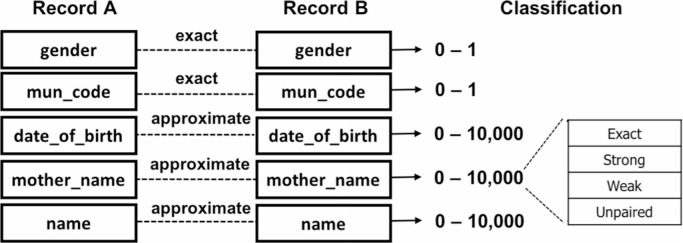
Hybrid linkage approach based on bespoke rules.

The second approach is a *hybrid* mixture of deterministic and probabilistic rules applied to individual linkage attributes (see Fig. 4). Categorical attributes are matched exactly, whereas names and dates (both more prone and sensitive to errors) are probabilistically classified as: exact (Dice = 10 000), strong (10 000 > Dice >= 9000), weak (9000 > Dice >= 8000), and unpaired (8000 > Dice). This approach results in some flexibility on the combinations of exact and approximate comparisons.

These methods produce three output data sets: true positive (TP) pairs, true negative (TN) pairs, and “dubious records” (FP and false negative (FN) matches). This classification is based on upper and lower cutoff points representing boundaries for TP and TN matches, respectively. We perform an analysis on which cutoff points retrieve more true (positive and negative) pairs and perform an iterative second round over dubious pairs, shifting these points in each iteration, to retrieve additional records into these two groups.

### C Accuracy Assessment

Resulting data sets produced by AtyImo are evaluated based on sensitivity, specificity, and PPV. We perform a manual review over samples with an incremental size from controlled databases (known coexistence of matching records) used as gold standards. This process allows us to quantify AtyImo’s accuracy, especially with regards to the choice of cutoff points that minimize the amount of false pairs. Additionally, it enables our approach to scale up to bigger databases (as in our case study), where a manual review is impossible or impractical.

Current Dice indices used as upper and lower cutoff points are 9.400 and 8.800, respectively. We chose the cutoff points after several iterative tests with different samples of variable size and data quality extracted from different databases. These tests aimed to check the variation on indices providing better results and the possibility of using the same values for all linkage executions. Table I summarizes one of our results obtained with four cohort samples linked to hospital episodes (SIH) and disease notification (SINAN) databases. We observed Dice values providing a better accuracy varying between 9.100 and 9.400, which highlights the challenges of trying to establish a default value.

**Table I:** Variability of Best Dice Coefficients

Samples	SIH			SINAN		
	Dice	Sens.	PPV	Dice	Sens.	PPV
SE	9400	95.6%	95.0%	9300	96.7%	95.9%
SC	9100	99.0%	96.0%	9100	97.7%	97.4%
BA	9100	98.5%	97.9%	9200	95.7%	95.5%
RO	9300	94.1%	94.2%	9400	87.9%	91.0%

We have started testing machine learning methods to design an automated accuracy checker and automatically retrieve dubious records. As we need to scale up to 114 million records, we expect this approach to help us in eliminating the need for a manual review and to efficiently deal with the variability of Dice values. Some preliminary results are discussed in [35].

## IV Case Study: The 114 Million Cohort

The Brazilian 114 million cohort [36] is a joint Brazil–U.K. effort started in 2013 with the aim of building a population-based cohort to enable diverse research studies on disease epidemiology and surveillance. The cohort was constructed based on data from CadastroÚnico (CADU database), a central register for individuals intending to participate in more than 20 social and protection programs kept by the Brazilian government. Bolsa Famllia (PBF database) is one of these programs and provides conditional cash transfers to families considered poor or extremely poor. So far, the cohort is comprised of 114 million individuals who have received payments from Bolsa Famflia between 2007 and 2015. This cohort is linked to public health databases to generate disease-specific data used in epidemiological studies.

Linkages between CADU and databases from social programes (including PBF) are deterministic, based on the NIS number—a unique identifier similar to a social security number. Linkages between the cohort and public health databases (the main ones are summarized in Table II) are performed probabilistically, as there are no common key identifiers across these databases. We developed AtyImo to enable us to perform these linkages in an accurate fashion.

**Table II:** Governmental Databases

Databases	Coverage
CADU (socioeconomic data)	2007 to 2015
PBF (cash benefits payments)	2007 to 2015
SIH (hospitalizations)	1998 to 2011
SIM (mortality)	2000 to 2012
SINAN (notifiable diseases)	2000 to 2010
SINASC (live births)	2001 to 2012

## V Accuracy and Scalability Results

Our evaluation strategy to assess AtyImo’s accuracy and scalability was based on some small size, controlled databases (where the number of matching pairs was known), as well samples from the CADU cohort with increasing sizes and variable data quality. We calculated accuracy metrics for each case and used ROC curves to visualize which cutoff points are the best similarity threshold discriminating matching pairs. Depending on sample sizes, we additionally performed a manual review for checking the results obtained and their accuracy.

AtyImo is implemented over Spark and over heterogeneous (CPU+multi-GPU) architectures. Synthetic data sets and both implementations are publicly available.^1^ The Spark-based implementation is structured as nine Python modules summarized in Table III. The *correlation()* module is the most time-consuming as it performs pairwise comparisons, similarity calculations, and matching decisions.

**Table III:** AtyImo-Spark Code Organization

Module	Purpose
*preprocessing.py createBlockKey.py* and *writeBlocks.py encondingBlocking.py correlation.py dedupByKey.py* and *createDatamart.py config.py* and *configstatic.py*	Data cleansing and standardization Blocking (record grouping)
	Creation of Bloom filters Pairwise comparison and matching Generation of research datasets
	Data and Spark configuration

### A Accuracy in Controlled Scenarios

Table IV presents a comparative analysis linking a controlled database with positive tests for rotavirus (children treated for diarrhoea) to a database with children’s hospital admissions for all-cause diseases (including diarrhoea). The first database had 486 records, to which we added 200 additional random records as noise. The second database had 9678 records. The goals were to correctly retrieve all 486 records from the second database (simulating a controlled behavior) and compare AtyImo’s results against other tools.

**Table IV:** Comparative Analysis–AtyImo × FRIL × Febrl

	FRIL	FRIL blocking	Febrl	Febrl blocking	AtyImo	AtyImo blocking
TP	486	484	480	479	486	486
TN	0	0	0	0	0	0
FP	1	0	1	0	0	0
FN	0	2	6	7	0	0

We observed similar accuracy in terms of TP and TN pairs, with a slight advantage for AtyImo when considering FP and FN pairs. We used the same comparison strategy for FRIL and Febrl: attributes *name* and *mother_name* were compared through the Jaro-Winkler distance (weight = 1), date difference for *date_of_birth* (weight = 0.9), exact match for *municipality_ code,* and *gender* (weight = 0.8 for both). This configuration is similar to AtyImo’s hybrid approach. Blocking was based on the *sorted neighborhood algorithm,* which sorts records through a given key and only compares records within a predefined distance window, whereas, for AtyImo, we used the predicate described in Section III. As FRIL and Febrl have a black-box implementation, we were unable to fully explore how blocking influences the results obtained.

### B Accuracy in Uncontrolled Scenarios

While the cohort creation was taking place, we performed experiments linking isolated CADU samples (from 2007 to 2015) to health databases covering specific diseases (e.g., tuberculosis, children mortality, BCG vaccination, etc.). Table V presents linkage results for tuberculosis between the CADU 2011 (best quality sample), the hospitalizations (SIH), and the disease notifications (SINAN) databases. We used samples from two Brazilian states: Sergipe (SE), the smallest sample (few individuals in CADU), and Santa Catarina (SC), a middle size sample. They were chosen for manual review purposes.

**Table V:** Linkage Results (sample: CADU Tuberculosis 2011)

Databases (number of records)	Matched pairs		TPs (%)	
	Full	Hybrid	Full	Hybrid
CADU 2011 × SIH SE	40	24	23	23
(1, 447 512) × (49)			(57.5%)	(95.8%)
CADU 2011 × SIH SC	140	95	83	86
(1 988 599) × (330)			(59.2%)	(90.5%)
CADU 2011 × SINAN SE	398	311	309	299
(1 447 512) × (624)			(77.6%)	(96.1%)
CADU 2011 × SINAN SC	661	500	551	462
(1 988 599) × (2049)			(83.3%)	(92.4%)

The hybrid approach retrieved more TP pairs compared to the full probabilistic routine, which emphasizes that individual comparison of linkage attributes provides more accurate results less influenced by imputation errors. We made similar tests with a bigger sample (BA) and a poorest data quality sample (RO) (see Table I).

**Fig. 5:**
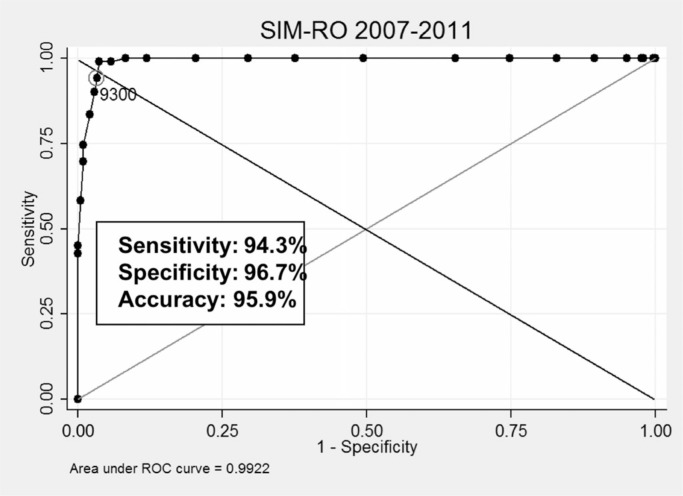
Best coefficient and related results (CADU cohort × SIM, RO).

**Fig. 6:**
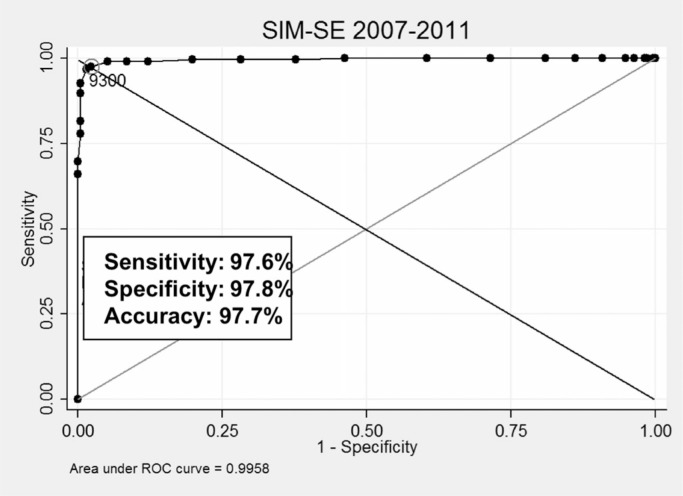
Best coefficient and related results (CADU cohort × SIM, SE).

**Fig. 7:**
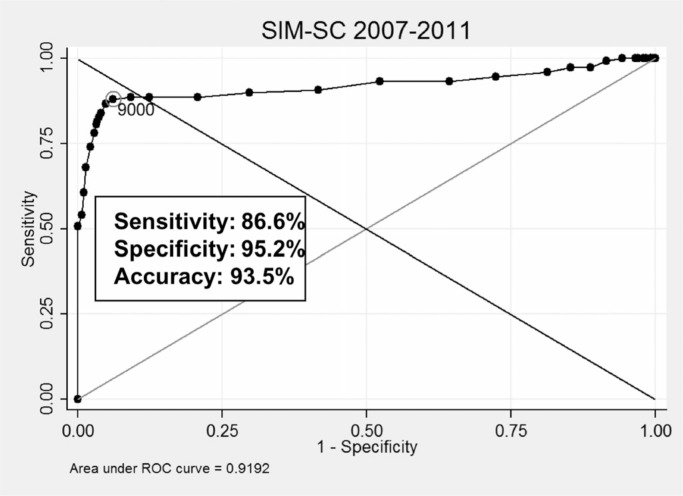
Best coefficient and related results (CADU cohort × SIM, SC).

In [5], we presented the overall cutoff points providing better results when linking cohort records to different mortality (SIM) samples (RO, SE, and SC), respectively: 9.300 (sensitivity 94.3%, PPV 95.9%), 9.300 (sensitivity 97.6%, PPV 97.7%), 9.000 (sensitivity 86.6%, PPV 93.5%). We plotted ROC curves for all experiments to visually assess the power of discrimination of each coefficient, as depicted in Figs. 5 to 7. Results from the SC sample were slightly worse compared to other samples, having been influenced by expressive missing data present in 2007 to 2009 fragments.

From these experiments, we observed which Dice values provided the best results for each case and measured the distance between them to verify the suitability of using the same coefficients for all linkages. Best coefficients varied from 8.800 to 9.400, being used as thresholds to separate dubious records. This observed variation reinforces the complexity of running probabilistic linkages without gold standards.

### C Scalability Evaluation

We measured the time spent on linkage for each tool in Table IV. Average times (in seconds) for five executions were: FRIL (681), Febrl (3.780), AtyImo (103); decreasing to FRIL (37), Febrl (2.730), AtyImo (42) using blocking. Although these results were obtained with a small database, they illustrate how AtyImo performs as good as other tools. We consider AtyImo’s major advantage as its ability to scale upwards to huge databases, which we were unable to do with other tools. We linked the entire cohort to 370.000 records from SINAN in nine days using 20 nodes (40 2.8 GHz cores, 256 GB RAM) from a dedicated supercomputer. We also linked 7 million cohort records to one million records from SIM in four days using a 56-core (3.1 GHz, 512 GB RAM) server.

Considering the potential speed up of parallel architectures, we have ported AtyImo to heterogeneous (CPU+GPU) platforms aiming to simultaneously use all available processors to distribute data and tasks. We have used a static strategy to assign data and tasks over available CPU and GPU subsystems. We initialize the runtime with as many CPU threads as CUDA devices, since one CPU thread is linked to each GPU to perform memory and control operations, plus a number of CPU threads linked to each CPU core to perform multicore computation. Each group of processing elements executing a computational kernel is seen as a combined processing unit, since CPU and GPU threads work in a co-ordinated fashion.

Scalability tests were performed for the *correlation* function since it is the most time-consuming component within the pipeline. Files to be linked are loaded in two matrices with one line per record. We exploit parallel matrix calculation and perform summation by partitioning the outermost loop into independent, variable size chunks, which allow us to better distribute the workload. Algorithm 1 shows the parallel version of AtyImo, where *cpu_exec()* and *kernel()* correspond to CPU and GPU versions, respectively, of the *correlation.py* module.

**Algorithm 1:**
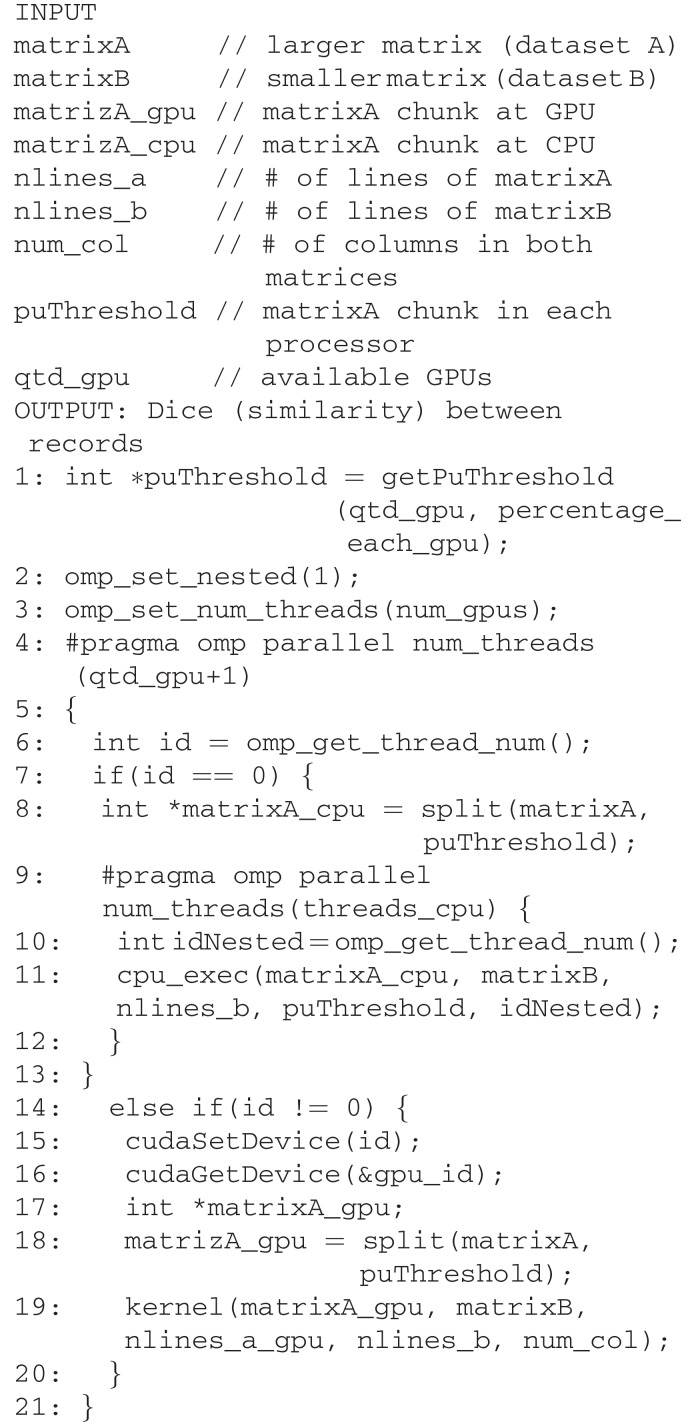
AtyImo code using OpenMP and CUDA.

**Fig. 8:**
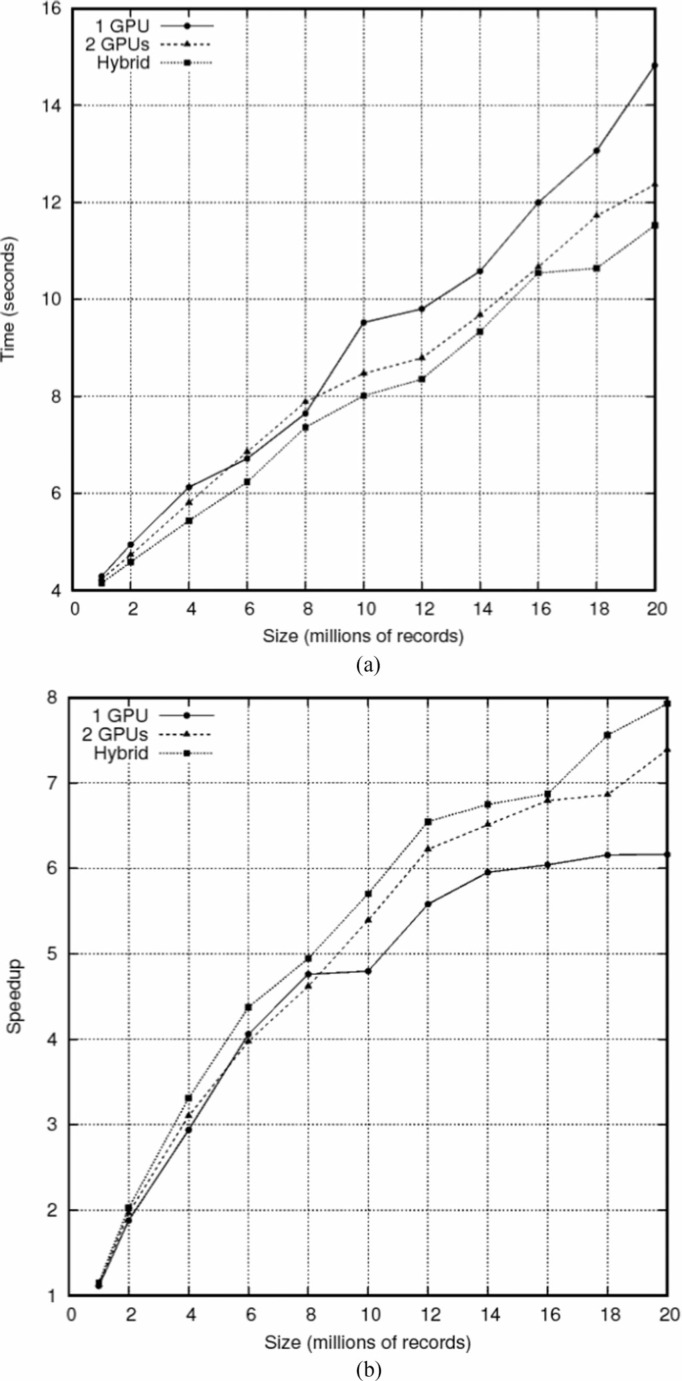
Execution time (a) and speed up (b) of AtyImo hybrid.

Fig. 8 illustrates the execution time and speed up obtained with samples varying from 1 to 20 million records linked using one or two GPUs, as well hybrid CPU+GPU cores. Speed up was calculated based on the CPU cores subsystem. The maximum speed up was around 8 for the hybrid subsystem, with a sustained ability to scale up to 20 million records. Our platform comprised of four Intel Xeon processors (3.33 GHz, 100 GB RAM, 6 cores, and 128 MB cache each) and two Tesla C2070 GPUs (448 cores in total). We used CUDA version 7.5.

## VI Conclusion

In this paper, we described and evaluated our probabilistic linkage approach implemented by AtyImo through accuracy and scalability results achieved in controlled and uncontrolled experiments. We analyzed accuracy metrics and ROC curves to identify effective similarity indices to generate high-accurate data for epidemiological studies. The variability of best Dice indices emphasized the complexity regarding the definition of gold standards for probabilistic linkage. AtyImo has proved to be very accurate linking controlled and uncontrolled databases. Its major contribution is the ability to link huge databases within a reasonable execution time and with a good accuracy.

We are working on improvements that will enable AtyImo to operate for the entire cohort over GPU architectures and designing machine learning methods to automatic accuracy assessment. We consider a careful discussion on the quality of data linkage as essential, particularly when large databases are used and mismatches might have important consequences for statistical analyzes in terms of bias.

## Footnotes

1 https://github.com/spiros/atyimo
